# Natural transmission of *Leishmania infantum* through experimentally infected *Phlebotomus perniciosus* highlights the virulence of *Leishmania* parasites circulating in the human visceral leishmaniasis outbreak in Madrid, Spain

**DOI:** 10.1186/s13567-015-0281-1

**Published:** 2015-12-09

**Authors:** Inés Martín-Martín, Maribel Jiménez, Estela González, César Eguiluz, Ricardo Molina

**Affiliations:** Unidad de Entomología Médica, Servicio de Parasitología, Centro Nacional de Microbiología, Instituto de Salud Carlos III, Ctra. Majadahonda-Pozuelo s/n, Majadahonda, 28220 Madrid, Spain; Unidad de Veterinaria, Centro Nacional de Microbiología, Instituto de Salud Carlos III, Ctra. Majadahonda-Pozuelo s/n, Majadahonda, 28220 Madrid, Spain

## Abstract

A human leishmaniasis outbreak is occurring in the Madrid region, Spain, with the parasite and vector involved being *Leishmania infantum* and *Phlebotomus perniciosus* respectively. The aim of this study was to investigate the virulence of *L. infantum* isolates from the focus using a natural transmission model. Hamsters were infected by intraperitoneal inoculation (IP) or by bites of sand flies experimentally infected with *L. infantum* isolates obtained from *P. perniciosus* collected in the outbreak area (IPER/ES/2012/BOS1FL1 and IPER/ES/2012/POL2FL6) and a well characterized *L.* *infantum* strain JPCM5 (MCAN/ES/98/LLM-877). Hamster infections were monitored by clinical examination, serology, culture, parasite burden, Giemsa-stained imprints, PCR, histopathology and xenodiagnostic studies. Establishment of infection of *L. infantum* was achieved with the JPCM5 strain and outbreak isolates by both *P. perniciosus* infective bites or IP route. However, high virulence of BOS1FL1 and POL2FL6 isolates was highlighted by the clinical outcome of disease, high parasite detection in spleen and liver, high parasitic loads and positivity of *Leishmania* serology. Transmission by bite of POL2FL6 infected flies generated a slower progression of clinical disease than IP infection, but both groups were infective to *P. perniciosus* by xenodiagnosis at 2 months post-infection. Conversely, hamsters inoculated with JPCM5 were not infective to sand flies. Histopathology studies confirmed the wide spread of POL2FL6 parasites to several organs. A visceral leishmaniasis model that mimics the natural transmission in nature allowed us to highlight the high virulence of isolates that are circulating in the focus. These findings contribute to a better understanding of the outbreak epidemiology.

## Introduction

Visceral leishmaniasis (VL) is considered one of the most devastating neglected diseases accounting worldwide with 58 000 cases per year [1]. The causative agent in the Mediterranean Basin is *Leishmania infantum* being promastigote forms transmitted to the vertebrate host by the bite of infected sand flies [2]. Animal models are essential to test new treatments and vaccines to fight this disease. In cutaneous leishmaniasis (CL), rodents have been widely used to reproduce skin lesions [3]. However, in the case of VL, mice do not completely reflect the human development of the disease and even infection in susceptible mice strains to *L. infantum* may become chronic. Therefore, the hamster is considered a better model to reproduce the clinicopathological features of human VL [3] and has been widely used for immunological studies [4–7]. A proper and reliable animal model should mimic the natural transmission conditions with special emphasis on dose, pathogen stage delivered, co-administered molecules and administration route [8]. In the case of *Leishmania,* transmission occurs when an infected sand fly takes or attempts to take a blood meal from the vertebrate host. This is a complex process in which sand flies, as telmophagic insects, need to lacerate tissues and create a hemorrhagic pool from where they ingest blood.

Arthropod-borne pathogens have adapted their routes of entry into the vertebrate hosts. The administration route determines the first encounter of the parasite with the immune system, which can drive either to a susceptible or protective response and is highly involved in the outcome of cutaneous or visceral disease. Therefore, infections initiated by parasites directly delivered into the bloodstream do not reproduce the immunological responses that take place under natural conditions [8].

The required number of parasites to initiate a *Leishmania* infection seems to depend on the *Leishmania* species or even the strain. Natural infectious doses are much lower than the experimental infectious doses used for parasite infection in the laboratory where 10^8^ or 10^7^ promastigotes are usually inoculated. The average of *L. infantum* parasites ejected from *Lutzomyia longipalpis* was recently established at 1000 promastigotes by Rogers et al., who analyzed it with feeding infected sand flies through chick skin [9]. Natural transmission models have provided information regarding individual variability. Concretely, the number of inoculated parasites in the *Leishmania major*-*Phlebotomus duboscqi* combination follow a bimodal distribution, corresponding to a low dose of less than 600 parasites, and a high dose between 1000 and 100 000 parasites [10]. Other authors have offered similar results and demonstrated that sand flies infected with a dermotropic strain inoculate a greater number of parasites than sand flies infected with a viscerotropic strain [11]. Moreover, *Leishmania* ejected doses do not seem to depend on the nature of the vertebrate hosts [12].

*Leishmania* parasites are inoculated into the dermis of the host along with very active immunomodulatory substances such as saliva and promastigote secretory gel (PSG). Sand fly saliva counteracts vertebrate hemostatic and immune responses to ensure blood feeding success and has been directly involved in the establishment of infection [13, 14]. On the contrary, PSG blocks the anterior part of the midgut and stomodeal valve. As a consequence, metacyclic promastigotes are regurgitated during blood feeding, resulting in an improved transmission efficacy [15].

In leishmaniasis animal models, cultured promastigotes are normally used as the source of infective material which is not the appropriate pathogen stage delivered in nature. Among several promastigote forms present in the midgut of sand flies only metacyclic stages are known to be resistant to vertebrate complement destruction and therefore able to initiate infection [16]. This drawback is usually overcome by working with stationary phase cultures which are enriched in metacyclic forms or isolation of metacyclic promastigotes by lectin agglutination [17].

Reports of laboratory transmission of *Leishmania* spp. by experimentally infected sand flies are still scarce. Success of natural transmission has been achieved mainly in CL models [14, 18–25]. Although VL models initiated through infected sand fly bites are not easy to reproduce, several studies have been conducted using mice, hamsters, rats or dogs as vertebrate hosts demonstrating succesful transmission [11, 12] or both succesful transmission and animal infection follow-up [26–33].

Human leishmaniasis due to *L. infantum* is endemic in Spain. However, there has been an unusual rise of human leishmaniasis cases in an urban area of the south-western Madrid region since 2010 [34]. A total of 616 human cases have been reported from the beginning of the outbreak to February 2015, corresponding to 38% VL. The large number of human cases has led to an increased incidence from 2.44/100 000 inhabitants in 2009 to 49.0/100  000 inhabitants in 2014 in Fuenlabrada, the most affected municipality (Community of Madrid, personal communication). On the contrary, canine leishmaniasis prevalence in the focus is lower than the average in the Madrid region [35, 36] and two wild animals—the hare and the rabbit- have shown to play a role as reservoirs [37, 38].

In this work, our aim was to study the virulence of *L. infantum* isolates that are circulating in the human leishmaniasis outbreak of Madrid using a natural transmission model of VL in the hamster.

## Materials and methods

### Parasites

*Leishmania infantum* parasites used in this study included two isolates from *Phlebotomus perniciosus* collected in the field during the entomological survey carried out in the transmission season of 2012 in the human leishmaniasis outbreak area in Madrid (IPER/ES/2012/BOS1FL1 and IPER/ES/2012/POL2FL6, referred to as BOS1FL1 and POL2FL6, respectively). Simultaneously, the well characterized *L.* *infantum* strain JPCM5 was used as a control. JPCM5 is a clone obtained from the JPC strain isolated from a naturally infected dog (MCAN/ES/98/LLM-877) and maintained at the WHO Collaborating Center for Leishmaniasis, Parasitology Department, Instituto de Salud Carlos III (ISCIII), Madrid, Spain. This clone was used for *L. infantum* genome sequencing [39] and has been widely utilized for experimental challenge [40].

All parasites were passaged through hamsters before the experiments. Promastigotes were cultured at 27 °C in NNN and RPMI supplemented with 10% inactivated fetal calf serum (FCS, Lonza, Basilea) and a mixture of penicillin and streptomycin (10 000 U/mL, Lonza, Basilea).

### Experimental infection of sand flies

A *Phlebotomus perniciosus* colony originally established with specimens collected in an endemic area of leishmaniasis in Madrid, Spain [41] maintained at 27 °C and 17:7 light-darkness photo-period at the insectary of the Medical Entomology Unit of the ISCIII was used. Three to six-day old female sand flies were fed on a mixture of defibrinated rabbit blood and 2.5 × 10^8^ or 2.5 × 10^7^/mL cultured promastigotes of JPCM5 strain or POL2FL6 isolate through a feeding device of chick skin membrane. Blood-fed female sand flies were separated and kept in a cage and after 5 days flies were transferred to oviposition pots. Some sand flies were dissected in order to evaluate the midgut infection development. Five days later which corresponded to 10 days after experimental infection, sand flies were allowed to take a second blood meal on hamsters to initiate parasite transmission. A total of four experimental infections of *P. perniciosus* with *L. infantum* promastigotes were conducted including two experimental infections with JPCM5 strain and two other with the POL2FL6 isolate.

### Animal infections

**Figure 1:**
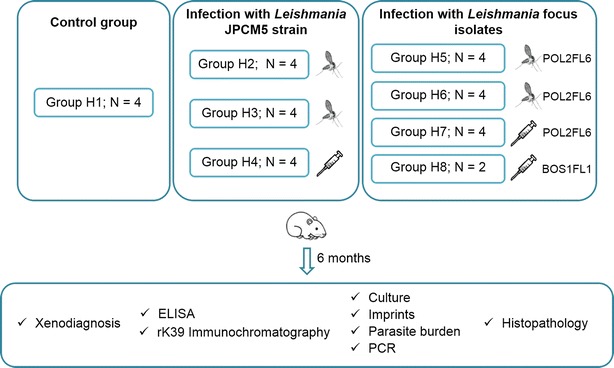
**Working scheme of animal infections.** Hamsters were split into eight groups including a control group of non-infected animals (Group H1), groups of hamsters infected with JPCM5 strain either by infective sand fly bites or by IP inoculation (Groups H2-H4) and hamsters infected with *L. infantum* isolates from the Madrid outbreak either by infective sand fly bites or by IP inoculation (Groups H5-H8).

Six-week-old goldhamsters (*Mesocricetus auratus*, strain RjHan:AURA, Janvier, France) were housed at the animal facilities of the ISCIII. Animal handling was carried out according to the standards specified in the Guide for Care and Use of Laboratory Animals and approved by the ethics committees for animal care and experimentation of the ISCIII (CBBA/4.2-PA 225/08). A total of 30 hamsters were divided into eight groups. They include four subgroups that were infected by natural transmission either with JPCM5 or POL2FL6 parasites, two subgroups of hamsters that were intraperitoneally inoculated with 10^7^ cultured promastigotes of JPCM5 strain and POL2FL6 isolate and a control group (uninfected). Additionally, two hamsters (Group H8) were inoculated with 10^7^ cultured promastigotes of BOS1FL1 isolate (Figure 1). For sand fly transmission, animals were anaesthetized with 150 mg/kg ketamine by subcutaneous route and individually placed in cages containing an average of 36 (SD = 18) potentially infected sand flies, depending on the survival rate after oviposition for each sand fly infection set (Table 1). During the exposure time, a person was checking and scoring for sand fly host landing, probing or successful blood-feeding. The site of blood-feeding was recorded to detect possible skin lesions in the area. After 1 hour, blood-fed female sand flies were dissected and their guts checked microscopically at 400× to confirm the presence of *Leishmania* promastigotes. The number of bites or attempts of bites received by each animal was documented.

**Table 1:** Experimental infections of *P. perniciosus* with *L. infantum* parasites.

Nº experiment	*Leishmania* strain or isolate	Infective mixture (parasites/mL)	Fed flies (%)^a^	Flies transfered to pots (%)^b^	Surviving flies after oviposition (%)^c^	Positive flies (%)^d^
1	JPCM5	2.5 × 10^8^	395/500 (79.0)	332/395 (84.1)	220/332 (66.3)	72/100 (72.0)
2	JPCM5	2.5 × 10^8^	425/500 (85.0)	216/425 (50.8)	196/216 (90.7)	88/96 (91.7)
3	POL2FL6	2.5 × 10^7^	317/500 (63.4)	240/317 (75.7)	150/240 (62.5)	18/21 (85.7)
4	POL2FL6	2.5 × 10^7^	303/500 (60.6)	151/303 (49.8)	17/151 (11.3)	15/17 (88.3)

^a^Blood-fed sand flies/exposed sand flies ×100

^b^Flies transferred to pots/blood-fed sand flies ×100

^c^Flies that survived oviposition/flies transferred to pots ×100

^d^ *Leishmania* positive sand flies/dissected sand flies ×100

### *Leishmania* infection follow-up

Clinical examination and weight were recorded weekly in all animals. Animals were anaesthetized with ketamine and euthanized by cardiac exsanguination at 6 months after challenge. Sera were collected and stored at −20 °C until use.

Animal necropsy was performed under sterile conditions. Liver and spleen were aseptically removed and weighed. These organs were cut into several portions that were used for the different protocols tested (culture and parasite burden, impression smears, histology or PCR).

### Culture, parasite burden and Giemsa-stained imprint preparation

Reserved portions of tissues were homogenized in NET-10 (10 mM Tris–HCl, 10 mM EDTA, 10 mM NaCl; pH 8.0) and passed through a cell strainer of 0.45 µm (Corning, Durham, NC). Cell suspension was cultured in NNN tubes at 27 °C that were checked weekly up to 4 weeks for promastigote detection under the microscope. Parasite burden was estimated by limit dilution assay [42]. Briefly, homogenized tissues were three-fold serially diluted with supplemented RPMI in microtiter culture plates containing a slant of NNN medium. Samples were cultured in duplicates and 24 dilutions were done. Plates were sealed and incubated at 27 °C and after 7 days, wells were examined for motile promastigote presence using an inverted microscope. Parasite burden was expressed as the number of parasites per mg of tissue, considering that the last positive dilution contained at least one living parasite. Impression smears were Giemsa-stained and examined at 1000X under the microscope.

### *Leishmania infantum* DNA detection by PCR

For PCR experiments, genomic DNA from 25 mg of spleen and liver tissues was extracted using the DNeasy^®^ Blood and Tissue Extraction Kit (QIAGEN, Hilden, Germany) according to the manufacturer’s instructions. *Leishmania* DNA was detected by kDNA PCR reaction which is based on the amplification of a 120 bp region of kinetoplastid DNA, as described elsewhere [43]. kDNA is an excellent target for *Leishmania* spp. detection that provides high sensitivity. Two hundred ng of DNA were used as the template and the following primers were used: JW11 (5′-CCTATTTTACACCAACCCCCAGT-3′) and JW12 (5′-GGGTAGGGGCGTTCTGCGAAA-3′). Reliability of the technique was ensured including negative and positive controls in all molecular steps. Negative controls consisted of PCR reaction tubes both without DNA or DNA belonging to non-infected animals (Group H1). As a positive control, 60 pg of genomic DNA extracted from JPCM5 *L. infantum* strain was used. PCR products were separated on Pronasafe 1.5% agarose gels (Conda, Spain) and visualized under a UV transilluminator.

### Xenodiagnosis

Infectivity of hamsters was assessed by xenodiagnosis as previously described [38]. Briefly, anaesthetized animals were individually exposed to 100 five to eight-day-old *P. perniciosus* females. Blood-fed sand flies were separated and maintained in adult cages under controlled conditions of humidity, temperature and photo-period for 5 days to allow transformation of potential amastigotes into promastigotes. Midgut dissections were carried out and sand fly infection status was determined by microscopy. Animals infected with JPCM5 strain and BOS1FL1 isolate were xenodiagnosed before sacrifice (6 months after challenge). In the case of 11 hamsters infected with POL2FL6 isolate, infectivity was thoroughly investigated and xenodiagnosis was performed at 2, 4 and 6 months.

### Histopathology of POL2FL6 infected hamsters

After necropsy, all tissues were formalin-fixed, and selected bone tissue was decalcified with acetic acid. Tissue Sections. (3 µm thick) were performed from salivary glands, stomach, small and large intestine, mesentery lymph nodes, heart, lung, kidney, adrenal glands, liver, spleen, brain, bone marrow, skin and genital tracts. In the case of skin, samples were taken from the abdomen. After hematoxylin and eosin staining, slides were examined for pathological lesions and the presence and quantification of *Leishmania* amastigotes. The parameters were scored as 0: no amastigote detection; 1: amastigotes hardly seen in localized areas; 2: more abundant presence of amastigotes, generally associated with other lessions such as granulomes or calcium agglomerates and 3: severe *Leishmania* parasitation.

### *Leishmania* serology

Specific anti-*L. infantum* IgG antibody response generated by the analyzed animals was measured by ELISA as described elsewhere [44]. Soluble *Leishmania* antigen (SLA) was prepared from *L. infantum* promastigotes (JPCM5 strain). Parasites were treated with lysis buffer (100 mM Tris–HCl, 1 mM EDTA, 1.6 mM PMSF and 0.5% protease inhibitor cocktail (Calbiochem); pH = 8) and disrupted by cavitation pump. Cell debris was spun down (27 000 g, 20 min followed by 100 000 g, 4 h) and the protein content of the supernatant was determined by the Bradford method. Plates were coated with 1 µg of SLA per well. Sera were diluted 1/200 in 0.1% BSA 0.3% PBS-Tween and peroxidase-conjugated goat anti-hamster IgG (1:2000, Southern Biotech, Birmingham, AL, USA) was used. The enzymatic reaction was developed by orthophenylendiamine (0.5 mg/mL) in McIlwein phosphate citrate buffer (pH 5.5) in the presence of 0.001% (v/v) of H_2_O_2_ (30%). Experiments were repeated at least twice. Serum dilutions from infected hamsters were considered positive when the mean of duplicate well values was greater than the cut off, calculated as the mean of the optical densisties (OD) from sera of 33 negative hamsters plus 3 times the standard deviation.

In addition, rK39 immunochromatographic tests (Kalazar Detect^®^ Human Rapid Test; InBios, Seattle, WA, USA) were performed with sera of hamsters following manufacturer’s recommendations.

### Statistical analysis

Analyses were done using Prism program version 5 (GraphPad Software, Inc., San Diego, CA, USA) and statistical significance was set as *p* value <0.05. Comparison between sets of groups was achieved by the non-parametric Mann–Whitney U test.

## Results

### Sand fly infections and transmission experiments

Each sand fly experimental infection was initiated with 500 *P. perniciosus*. The information regarding sand fly infection experiments including *Leishmania* strains, blood-feeding rates, number of sand flies transferred to oviposition pots, survival and infection rates is summarized in Table 1.

Experimental infections of *P. perniciosus* were achieved with infective mixtures of *L. infantum* promastigotes and rabbit blood at a final concentration of 2.5 × 10^8^ parasites/mL as a standard procedure in our laboratory. Infected flies with JPCM5 strain were successful in a second blood-feeding, midgut dissections showing that promastigotes had invaded the stomodeal valve and the presence of abundant metacyclic promastigotes was observed. For POL2FL6 isolate, infectious doses of 2.5 × 10^8^ resulted in such a heavy infection that sand flies were not able to take a second blood meal. Therefore, a final concentration of 2.5 × 10^7^ parasites/mL was chosen for transmission experiments with POL2FL6 isolate. *P. perniciosus* infections with POL2FL6 isolate were successful as determined by massive colonization of the complete midgut including a stomodeal valve with metacyclic promastigote presence. However, many massively infected flies with POL2FL6 were still unable to take blood from hamsters, leading to a lower number of bites in animals bitten by POL2FL6-infected sand flies when compared to animals bitten by JPCM5-infected sand flies (Table 2). When working with POL2FL6 isolate, three hamsters were bitten by an individual sand fly with confirmed blood meal under the microscope. Four hamsters received long time probing of infected flies without a successful blood meal. This situation was classified as attempts of biting. There was one hamster that did not receive any bite or attempt of biting (Group H6); therefore, it was excluded from the transmission experiment.

**Table 2:** Data of experimentally infected hamsters.

Group	Transmission route	*Leishmania* strain or isolate	No. of exposed sand flies^a^	No. of infective bites (%)^b^	Culture		Parasite burden^c^	
					Spleen	Liver	Spleen	Liver
H1	Control	X	X	X	–	–	–	–
			X	X	–	–	–	–
			X	X	–	–	–	–
			X	X	–	–	–	–
H2	Bite	JPCM5	55	14/55 (25.5%)	+	–	–	95
			55	13/55 (23.6%)	–	–	4 × 10^2^	–
			55	16/55 (29.1%)	–	–	–	–
			55	14/55 (25.5%)	–	–	–	–
H3	Bite	JPCM5	49	25/49 (51.0%)	+	–	–	–
			49	21/49 (42.9%)	–	–	–	–
			49	14/49 (28.6%)	–	–	–	–
			49	14/49 (28.6%)	–	+	–	3.1 × 10^3^
H4	IP	JPCM5	X	X	+	–	2.6 × 10^2^	–
			X	X	–	–	–	–
			X	X	–	–	–	–
			X	X	–	–	–	–
H5	Bite	POL2FL6	25	1 Attemp	–	–	–	–
			25	1 Attemp	–	–	–	–
			25	>5 Attemps	–	–	3.3 × 10^5^	2.9 × 10^4^
			25	3 Attemps	+	+	8.8 × 10^12^	2.0 × 10^10^
H6	Bite	POL2FL6	25	1/25 (4.0%)	+	–	6.5 × 10^5^	6.7 × 10^8^
			25	1/25 (4.0%)	–	–	1.3 × 10^4^	–
			17	1/17 (5.9%)	–	–	–	–
H7	IP	POL2FL6	X	X	+	+	2.3 × 10^13^	2.1 × 10^12^
			X	X	+	+	3.9 × 10^11^	5.3 × 10^10^
			X	X	+	+	2.4 × 10^13^	1.9 × 10^12^
			X	X	+	+	6.4 × 10^12^	5.9 × 10^11^
H8	IP	BOS1FL1	X	X	+	+	3.2 × 10^12^	9.3 × 10^10^
			X	X	+	+	6.3 × 10^13^	5.9 × 10^12^

^a^The number of sand flies used for *Leishmania* challenge depended on the amount of female flies that survived oviposition. Survival flies were divided into cages where animals were placed for challenge

^b^The number of infective bites that each animal received is calculated as the number of flies that took a blood meal and was shown to be infected by *Leishmania,* as checked under the microscope, divided by the number of exposed flies ×100

^c^Parasite burden is expressed as the number of parasites per gram of tissue

### Evolution of hamster infection with JPCM5 strain and outbreak isolates

**Figure 2:**
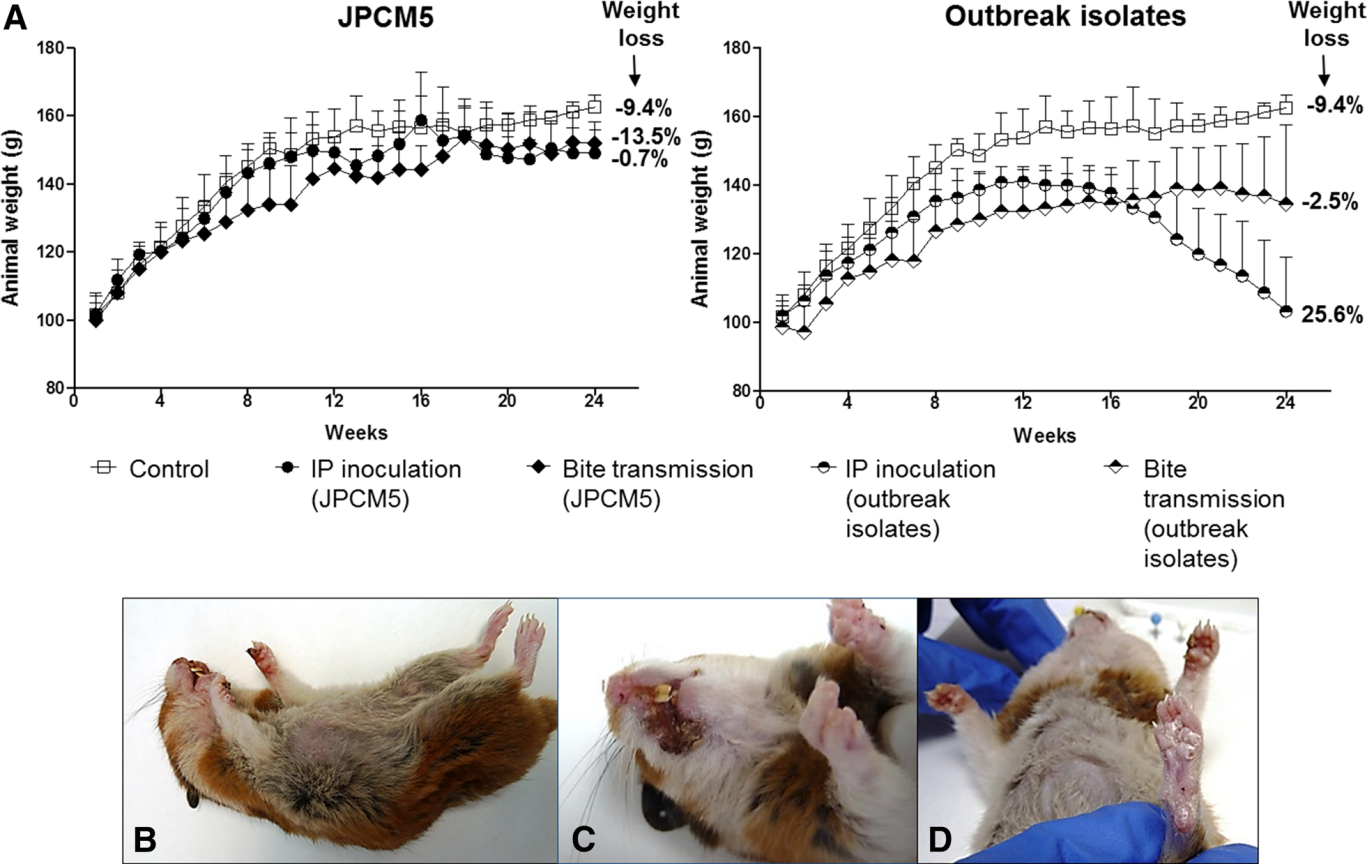
**Leishmaniasis follow-up over 24** **weeks.** **A** Animal weight follow-up. Bars represent standard deviation among animals within the same group. Weight loss is indicated in the right side of each plot. Weight loss was calculated using the following formula: 100 – [100*(weight average at week 10/weight average at week 24)]. A positive number indicates weight loss while negative numbers show weight gain; **B** General clinicopathologic features in representative sick hamsters infected with outbreak isolates. General condition; **C** Leishmaniasis skin lessions on mouth and **D** hindpaw of hamsters infected with POL2FL6.

Establishment of infection of *L. infantum* was achieved with JPCM5 strain (5/12 animals; 41.7%) and outbreak isolates (10/13 animals; 76.9%) by both routes of infection. Weekly clinical examination showed different degrees of severity according to *Leishmania* strain and infection route. Animals inoculated or transmitted by bite with JPCM5 strain did not show any clinical signs including weight loss during the post-infection period. Conversely, hamsters infected with POL2FL6 or BOS1FL1 isolates exhibited an apparent clinical worsening during the follow-up period, with the onset of the disease faster in the case of inoculated animals as noted by the severity of weight loss in some cases. Concretely, infected animals with outbreak isolates gained weight at a markedly slower rate than the control group. At approximately 10 weeks, animal weight reached a plateau after which infected animals with outbreak isolates sustained their weight or started to drop in the IP inoculated animals reaching weight-loss values of 25.6% (Figure 2A).

**Figure 3:**
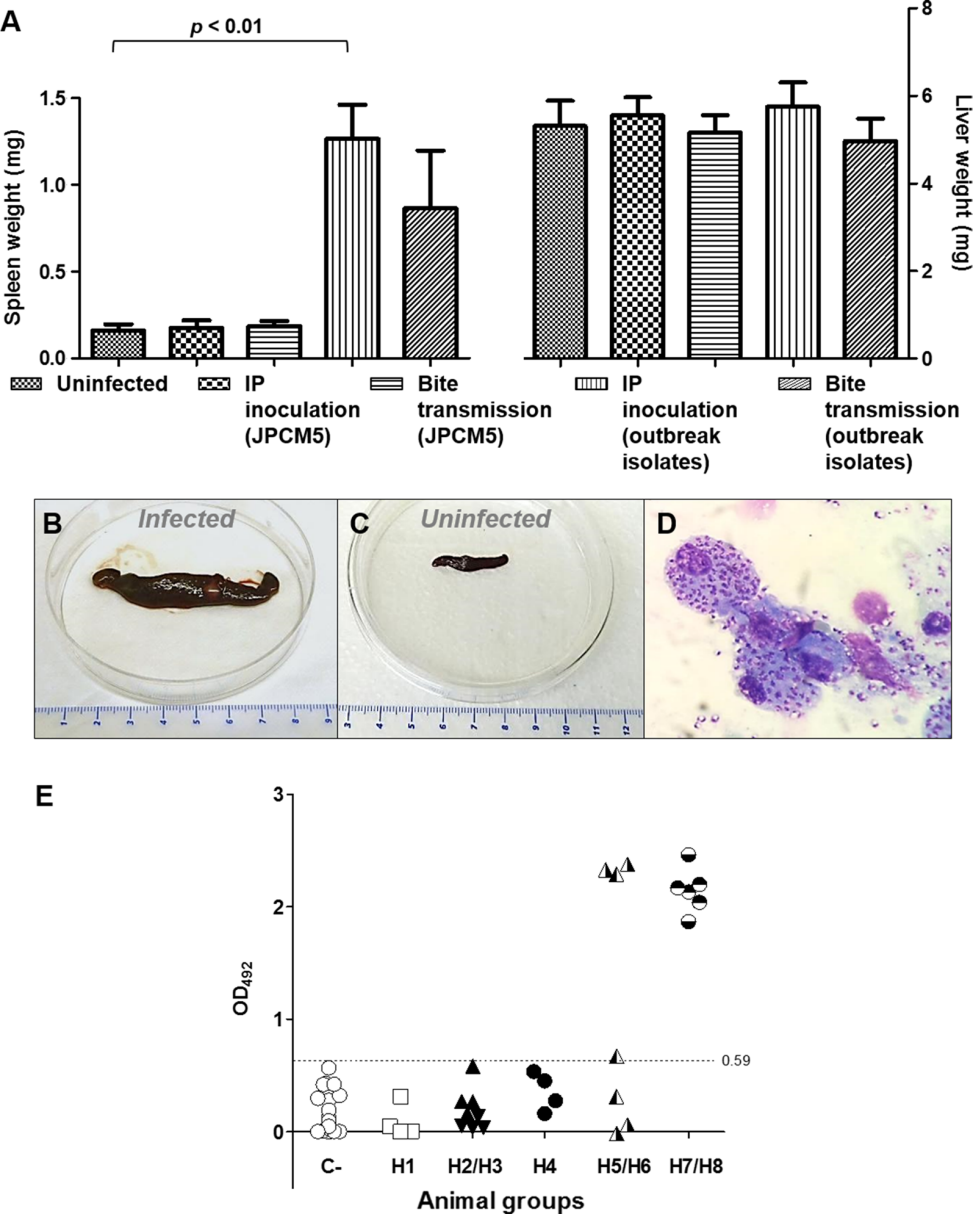
**Spleen and liver features and anti-** ***Leishmania*** **humoral response of infected hamsters. A** Weights of spleen and liver of animals infected with JPCM5 strain and isolates from the focus were compared with organ weights from uninfected hamsters according to the route of infection. Bars represent the standard deviation. *P* < 0.05 was considered statistically significant. Spleen of hamster infected by sand fly bites with POL2FL6 showing severe splenomegaly **(B)** compared to spleen of non-inoculated animal **(C)**. **D** Giemsa-stained imprint of spleen of hamster infected by sand fly bites with POL2FL6 showing macrophages packed with amastigotes. **E** Anti-*L. infantum* IgG antibody levels in sera of hamsters. ○: Control animals; □: Hamster group H1; : Hamster group H2; : Hamster group H3; : Hamster group H4; : Hamster group H5; : Hamster group H6; : Hamster group H7; : Hamster group H8; 0.59 OD cut off value was established with sera of non-infected hamsters.

Hamsters infected with outbreak isolates showed signs of infection such as skin scaling or appearance of skin lesions affecting paws, mouth and snout (Figures 2B, C, and D). At necropsy, no pathological findings were recorded in animals infected with JPCM5. On the contrary, most of the hamsters infected with the outbreak isolates presented severe splenomegaly when compared to spleens from uninfected animals (Figures 3A–C). Spleen was often found fibrotic with pale indurations (Figure 3B). Hepatomegaly was only observed in the most severe cases of hamsters infected with outbreak isolates, although the difference among groups was not statistically significant (Figure 3A).

In animals infected with JPCM5 strain, parasites were visualized in the spleen or liver of 41.7% of hamsters by imprints, culture and/or limit dilution assay yielding low parasitic loads. Kinetoplast DNA PCR confirmed the culture results. Conversely, *Leishmania* parasites were detected by culture, limit dilution assay, tissue imprints (Figure 3D) or PCR in most of the animals infected with the outbreak isolates (76.9%), which presented two-to-six fold greater parasitic loads than animals infected with JPCM5 (Table 2). In certain cases and using the same samples, parasites did not grow in cultures but were detected by limit dilution assay (i.e. H6 group) which could be a matter of better environmental growth conditions in the microtiter wells rather than in the 10 mL tubes.

Regarding *Leishmania* serology, the average readout of negative control hamsters sera was 0.12 and the cut off level was set at an OD of 0.53. None of the sera from hamsters infected with JPCM5 strain presented specific IgG against the *Leishmania* protein extract (SLA). Nevertheless, 69.2% of the animals infected with the outbreak isolates (Groups H5-H8) showed levels of anti-*Leishmania* antibodies above the cut off level (Figure 3E). This positive humoral response correlated with the direct diagnosis in most of the cases (88.9%). Immunochromatography tests (rK39) were conducted with hamster sera performing high specificity (100%) when compared to SLA ELISA results. None of the JPCM5 infected animals showed a positive rK39 test while 6/13 (46.2%) of hamsters infected with the outbreak isolates exhibited a positive rk39 test. Therefore, sensitivity decreased to 66.7% when compared to ELISA results. Faint recognition bands were observed even with sera that had shown high levels of anti-*Leishmania* antibodies in ELISA (i.e. H7.2: OD_492_ = 2.2; H8.1: OD_492_ = 2.5 and H8.2: OD_492_ = 1.9).

### Xenodiagnosis follow-up

**Figure 4:**
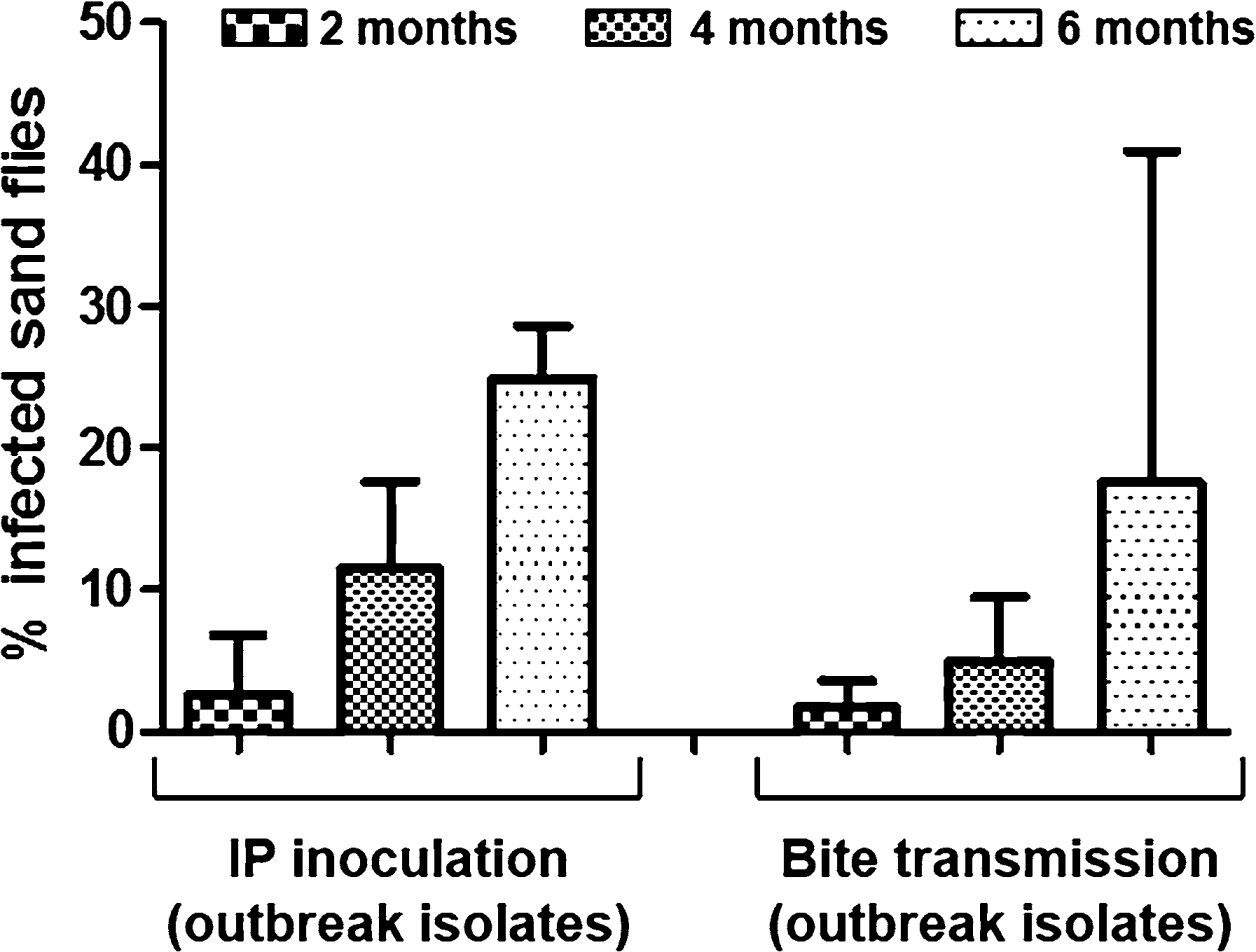
**Xenodiagnostic follow-up of hamsters infected with outbreak isolates.** Infectivity of infected hamsters was assessed by xenodiagnosis. Animals infected with JPCM5 were not infective for sand flies. Only xenodiagnosis of animals infected with outbreak isolates yielded positive results at 2, 4 and 6 months after IP inoculation or sand fly bite transmission. Bars represent the standard deviation.

**Figure 5:**
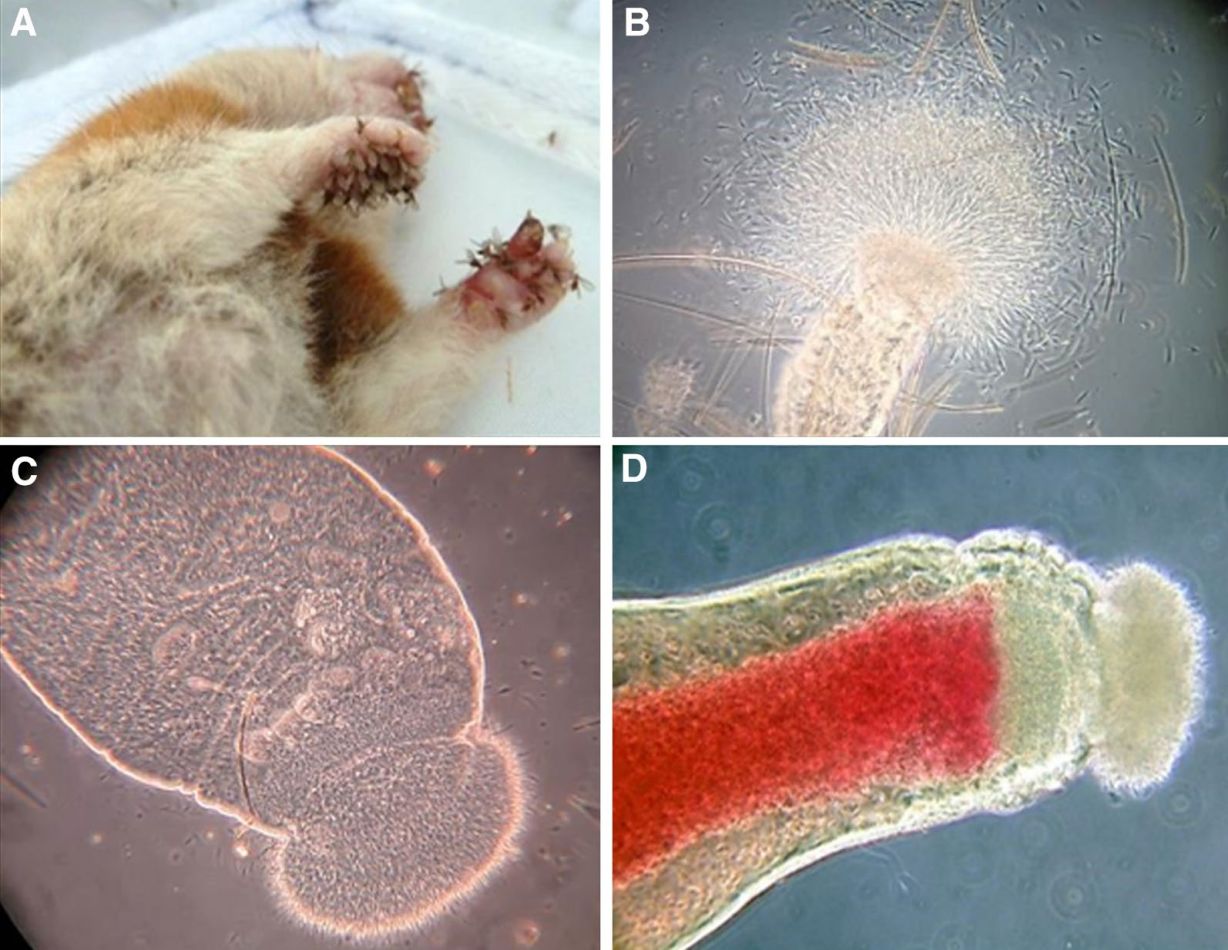
**Sand fly experimental infections and xenodiagnostic studies. A** Colonized *P. perniciosus* taking a blood meal from animal paws of anesthetized hamster during xenodiagnosis; **B** Midgut dissection of *P. perniciosus* infected with POL2FL6 5 days after xenodiagnosis, with motile promastigotes coming out of the anterior part; **C** Midgut of an infected sand fly showing an enlarged stomodeal valve; **D** Midgut dissection of experimentally infected *P. perniciosus* after biting on a hamster (re-feeding) and thus, potentially transmitting *Leishmania* parasites.

A total of 37 xenodiagnostic studies were carried out. Intraperitoneal inoculated hamsters with JPCM5 strain were not able to infect any sand fly by xenodiagnosis. Conversely, hamsters infected with the outbreak isolates yielded positive results in 100% of animals IP inoculated and 43% of animals transmitted by bites. In the xenodiagnostic follow-up study, 30% of the animals infected with POL2FL6 were infective to sand flies as soon as two months post-infection. The number of positive sand flies increased over the study timeline (2–4–6 months), reaching values of 44.4% in the case of a hamster transmitted with POL2FL6 by sand fly bites (Table 3; Figures 4 and 5).

**Table 3:** Xenodiagnosis of infected hamsters.

Hamster code	*Leishmania*	Transmission route	Time post-infection (months)	Engorged flies (%)^a^	Dissected flies (%)^b^	Positive flies (%)^c^	Parasites in s.v. (%)^d^
H4.1	JPCM5	IP	6	94/100 (94)	65/94 (69.1)	0	–
H4.2	JPCM5	IP	6	81/100 (81)	70/81 (86.4)	0	–
H4.3	JPCM5	IP	6	87/100 (87)	65/87 (74.7)	0	–
H4.4	JPCM5	IP	6	90/100 (90)	63/90 (70.0)	0	–
H5.1	POL2FL6	Bite	2	90/100 (90)	66/90 (73.3)	0/66 (0)	–
			4	81/100 (81)	73/81 (90.1)	0/73 (0)	–
			6	100/100 (100)	87/100 (87.0)	0/87 (0)	–
H5.2	POL2FL6	Bite	2	82/100 (82)	55/82 (67.1)	0/55 (0)	–
			4	81/100 (81)	64/81 (79.0)	0/64 (0)	–
			6	99/100 (99)	79/99 (79.8)	0/79 (0)	–
H5.3	POL2FL6	Bite	2	100/100 (100)	54/100 (54.0)	2/54 (3.7)	2/2 (100%)
			4	84/100 (84)	72/84 (85.7)	4/72 (5.6)	4/4 (100%)
			6	93/100 (93)	71/93 (76.3)	5/71 (7.0)	4/5 (80.0%)
H5.4	POL2FL6	Bite	2	87/100 (87)	67/87 (77.0)	1/67 (1.4)	1/1 (100%)
			4	88/100 (88)	77/88 (87.5)	7/77 (9.1)	7/7 (100%)
			6	99/100 (99)	81/99 (81.8)	36/81 (44.4)	33/36 (91.7%)
H6.1	POL2FL6	Bite	2	96/100 (96)	67/96 (69.8)	0/67 (0)	–
			4	46/100 (46)	39/46 (84.8)	0/39 (0)	–
			6	100/100 (100)	86/100 (86.0)	1/86 (1.2)	1/1 (100%)
H6.2	POL2FL6	Bite	2	90/100 (90)	67/90 (74.4)	0/67 (0)	–
			4	90/100 (90)	28/90 (31.1)	0/28 (0)	–
			6	95/100 (95)	69/95 (68.4)	0/69 (0%)	–
H6.3	POL2FL6	Bite	2	98/100 (98)	81/98 (82.7)	0/81 (0)	–
			4	99/100 (99)	89/99 (89.9)	0/89 (0)	–
			6	99/100 (99)	86/99 (86.8)	0/86 (0)	–
H7.1	POL2FL6	IP	2	92/100 (92)	53/92 (57.6)	4/53 (7.5)	4/4 (100%)
			4	92/100 (92)	33/92 (35.9)	3/33 (9.1)	2/3 (66.7%)
			6	100/100 (100)	83/100 (83.0)	21/83 (25.3)	5/21 (23.8%)
H7.2	POL2FL6	IP	2	72/100 (72)	28/72 (38.9)	0/28 (0)	–
			4	97/100 (97)	92/97 (94.8)	17/92 (18.5)	9/17 (52.9%)
			6	89/100 (89)	62/89 (69.7)	13/62 (21.0)	11/13 (84.6%)
H7.3	POL2FL6	IP	2	92/100 (92)	39/92 (42.2)	0/39 (0)	–
			4	71/100 (71)	59/71 (83.1)	4/59 (6.8)	3/4 (75.0%)
			6	91/100 (91)	81/91 (89.0)	23/81 (28.4)	19/23 (82.6%)
H7.4	POL2FL6	IP	6	96/100 (96)	72/96 (75.0)	3/72 (4.2)	0 (0%)
H8.1	BOS1FL1	IP	6	94/100 (94)	93/94 (98.9)	11/93 (11.8)	5/11 (45.5%)
H8.2	BOS1FL1	IP	6	97/100 (97)	83/97 (85.6)	15/83 (18.1)	6/15 (40.0%)

Metacyclic promastigotes were observed in s. v. of all sand flies examined

^a^Blood-fed sand flies/exposed sand flies ×100

^b^Dissected sand flies/total fed flies ×100

^c^ *Leishmania* positive sand flies/dissected sand flies ×100

^d^Maturity of infection is estimated as the percentage of sand flies with promastigotes in the stomodeal valve (s. v.)

### Histopathology

**Figure 6:**
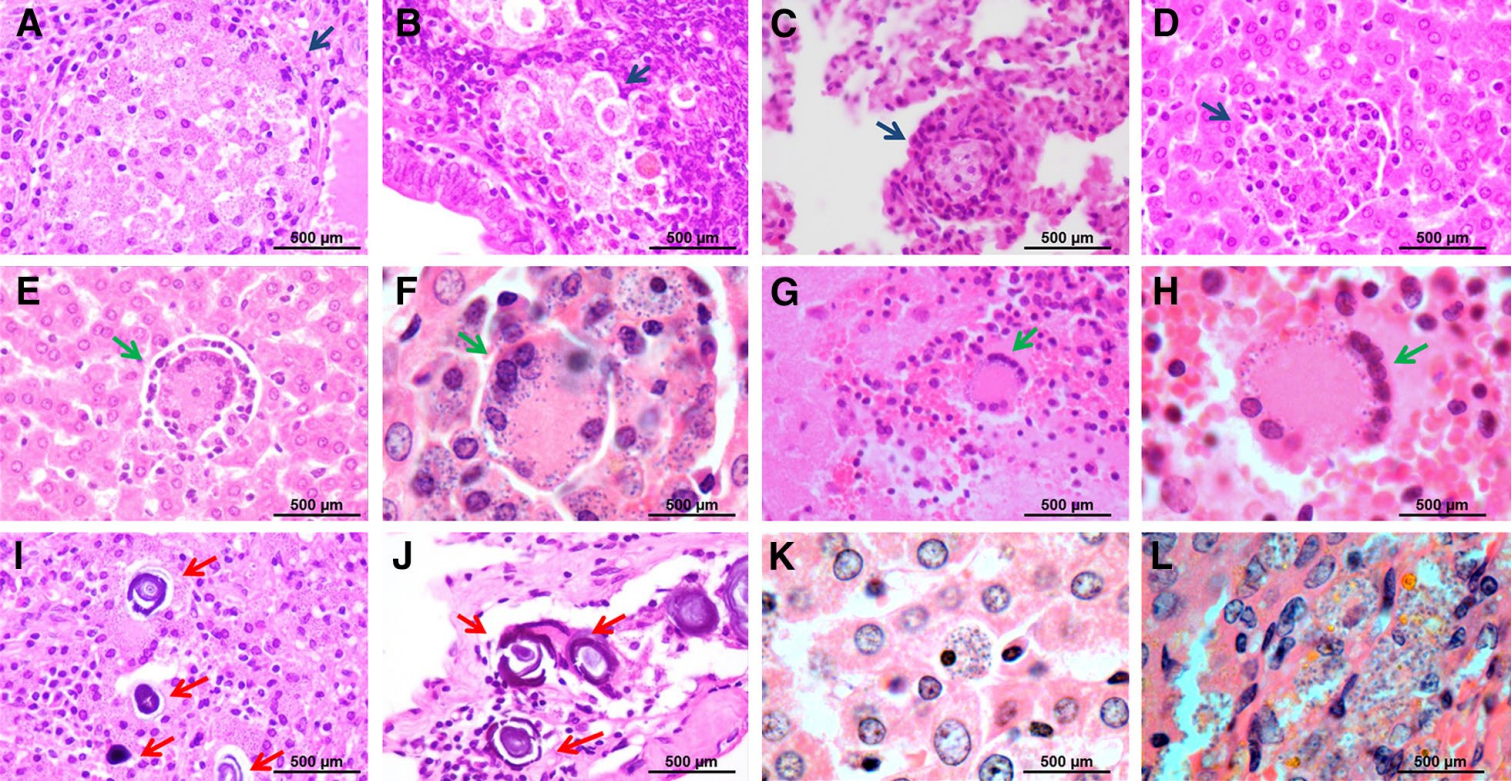
**Histological sections of selected tissues from infected hamsters.** **A**–**D** Granuloma in spleen, uterus, lung and liver (×400), respectively, highlighted with blue arrows; **E**–**H** Syncytial cells found in liver (×400 and ×1000) and brain (×400 and ×1000), respectively, indicated by green arrows. **I**–**J** Calcium deposition in liver and peritoneum, marked with red arrows. **K**–**L)** Liver and adrenal gland sections full of amastigote parasites (×1000).

*Leishmania* amastigotes were found in several tissues of hamsters infected with POL2FL6 isolate including salivary glands, stomach, small and large intestine, mesenteric glands, lung, kidney, adrenal glands, liver, spleen, brain, bone marrow, skin and reproductive organs of hamsters. Infected inflammatory cells full of amastigote forms within the cytoplasm were abundant in the liver, spleen, bone marrow and lymphoid tissues such as mesenteric glands or Peyer’s patches. Hepatic granulomas were present in all of the animals studied and even when amastigotes were not observed (Table 4). Granulomas and amastigote nets were also found in reproductive organs both in male and female hamsters. Amastigotes were visualized in prostatic interstice, epididymis interstice and epithelium of seminal vesicle of male inoculated animals. Moreover, granulomas with amastigotes were found in utero. Inoculated animals and some hamsters transmitted by sand fly bites showed a high degree of parasite dissemination (Table 4; Figure 6).

**Table 4:** Histopathology of several POL2FL6 infected hamsters.

Tissue	Negative control	H5.3	H5.4	H6.1	H6.2	H6.3	H7.1	H7.2	H7.3
Salivary glands	0	ND	1	0	ND	0	1	1	0
Stomach	0	ND	2	0	ND	0	2	2	1
Small intestine	0	ND	2	0	0	0	2	2	2
Large intestine	0	ND	1	0	0	0	1	1	1
Mesenteric ganglion	0	ND	3	0	0	0	3	3	3
Heart	0	ND	0	0	0	0	0	0	0
Lung	0	ND	1	0	0	0	1	1	1
Kidney	0	ND	0	1	0	0	1	1	1
Adrenal gland (cortex)	0	ND	2	0	0	0	1	1	1
Liver	0	0	3	0	0	0	3	3	3
Spleen	0	0	3	0	0	0	3	3	3
Pancreas	0	ND	0	0	0	0	0	0	0
Brain	0	ND	0	0	0	0	1	1	0
Bone marrow	0	ND	3	0	0	ND	ND	ND	ND
Skin	0	ND	1	0	0	0	ND	0	ND
Reproductive organs	0	ND	3	0	0	0	2	1	1

Negative control corresponds to a non infected hamster. H5.3, H5.4: Group 5, hamsters 3 and 4 (*Leishmania* vector-initiated infection). H6.1, H6.2, H6.3: Group 6, hamsters 1, 2 and 3 (*Leishmania* vector-initiated infection). H7.1, H7.2, H7.3: Group 7, hamsters 1, 2 and 3 (*Leishmania* intraperitoneal inoculation)

ND, not determined; 0, no amastigote detection; 1, amastigotes hardly seen in localized areas; 2, more abundant presence of amastigotes, generally associated with other lessions such as granulomes or calcium agglomerates and 3, severe *Leishmania* parasitation

Besides some degree of calcium deposition was observed in tissues from the non-infected hamster, agglomerates were frequently seen in most of infected analyzed tissues as a sign of infection and amastigote nests were usually found nearby.

## Discussion

The high virulence of two *L. infantum* isolates that are circulating in the Madrid LV outbreak was stressed using a hamster model. Establishment of infection of *L. infantum* was achieved with JPCM5 strain and outbreak isolates both by *P. perniciosus* infective bites or IP route. However, virulence of outbreak isolates was highlighted by the worse clinical outcome of disease, higher parasite detection in the spleen and liver, greater parasitic loads and positivity of *Leishmania* serology when compared with hamsters infected with JPCM5 strain.

Natural transmission models are crucial for studying immunological processes of leishmaniasis disease. The need for developing these models was clearly evident when vaccination protected mice against needle challenge but failed to protect animals against infected sand fly challenge [45, 46]. In this case, a natural *Leishmania* transmission model helped us to confirm the great virulence of outbreak isolates that was also observed with IP inoculation. However, a better standardization of the natural model is required for future use. More precisely, the number of parasites for sand fly infections should be titrated for sand fly infections and lower concentrations should be selected. Besides, implementing posttransmission scoring and metacyclic abundance determination would be useful to assess the rate of mature infection within a group.

Noteworthy, the entire *L. infantum* cycle was accomplished under laboratory conditions. Therefore, parasites originally isolated from collected sand flies in the field were used to infect hamsters throught the bite of experimentally infected colonized *P. perniciosus*. Morover, parasites were recovered by sand flies by xenodiagnosis.

Our results indicate that infection by syringe is more consistent than transmission by bite. In the case of the outbreak isolates, intraperitoneal inoculation leaded to severe clinical outcome in all animals (Groups H7 and H8). However, in animals infected by sand fly bites, disease development was more variable showing different degrees of clinical signs (Groups H5 and H6). This variability can be considered a more accurate reflection of what occurs in nature and it has already been described in hamsters infected with *Leishmania chagasi* by the bite of infected *L. longipalpis* [28]. This unpredictable outcome of clinical disease may be related to the individual variability of *Leishmania* doses ejected by infected sand flies [10, 11]. Furthermore, Kimblin et al. [10] correlated a rapid development of large lessions in the ears of mice that received the high-dose inoculum (5.6 × 10^3^ promastigotes).

Under laboratory conditions, natural transmission experiments are mainly hampered since the majority of sand flies die after oviposition in the laboratory [47]. Survival rates were improved by delaying to 5 days the time after blood-feeding before transferring the sand flies to oviposition pots. Experimental infections of *P. perniciosus* with both JPCM5 strain and POL2FL6 isolate resulted in heavy infections with metacyclic promastigotes blocking the stomodeal valve. However, several differences in terms of infection status were found. Sand fly infections with POL2FL6 isolate were massive; midguts were packed with great numbers of highly motile promastigotes and stomodeal valves were completely blocked. Those heavily infected sand flies were unable to re-feed but one bite or even attempts of biting without imbibing blood were sufficient for transmission with POL2FL6 isolate. *Leishmania* is known to modify sand fly biting behavior. Such interference in feeding behavior results in a greater number of bites by females attempting to take blood, thus favoring the transmission chances to a higher number of hosts in nature [15, 18]. Parasite transmission only by probing sand flies has been demonstrated for CL [18, 19, 47–49] as well as for VL [50].

We would like to remark that the differences found in midgut colonization should be considered for further investigation, since the massive colonization of outbreak isolates can determine the vector capacity. Moreover, massive colonization of midgut and stomodeal valve was also observed in natural infections from field sand flies collected in the outbreak area (unpublished observations).

Regarding *Leishmania* serology, in most of the cases infection with the outbreak isolates elicited a high titer of specific IgG. However, rk39 immunochromatography performed with the same sera resulted in a lower sensitivity compared to the ELISA tests (66.7%). Even sera showing high titers of IgG anti-SLA gave faint bands in the rk39 immunochromatography commercial tests. This might be due to the slight binding affinity of protein A to the Fc receptor of hamster IgG [51].

Due to the high virulence observed in POL2FL6 isolate a deeper insight into the dissemination process of these parasites in different vertebrate tissues was gained. Histopathology studies confirmed the wide spread of POL2FL6 parasites. Spleen, liver, bone marrow and lymph nodes were found highly parasitized. These organs are largely known to be involved in leishmaniasis pathology [52]. On the contrary, amastigote forms were found in other not so common or less studied locations such as salivary glands, stomach, intestine, mesenteric glands, lung, kidney, adrenal glands, brain and reproductive organs of the hamster. The presence of amastigotes in renal, gastrointestinal and respiratory systems has been described [52–54] and even in the central nervous system of experimentally infected hamsters and naturally infected dogs [55, 56]. Surprisingly, parasites were found in the skin of only one animal. Taking into consideration that hamsters infected with POL2FL6 isolate gave high rates of infected sand flies as evaluated by xenodiagnosis, visualization of parasites was expected in all skin samples. Future studies are planned to further investigate the dissemination of different parasite strains along the skin and its relationship with sand fly infectiousness.

Transmission by bite of POL2FL6 isolate generated a slower progression of clinical disease than IP infection providing a better reflection of the chronicity that takes place in nature. However, both groups were infective to *P. perniciosus* by xenodiagnosis as soon as 2 months post-infection. Conversely, hamsters inoculated with JPCM5 were not infective to sand flies. In our experiments, the capability to transmit *Leishmania* parasites from infected animals to sand flies is related to the disease progression. This relationship between clinical severity and proportion of infectious dogs has been widely reported [57]. Similarly, hamsters showing worse clinical conditions exhibited higher infectivity rates to sand flies. Moreover, this association was clearly demonstrated as the infectiousness of hamsters infected by POL2FL6 to sand flies increased over time reaching values of 44.4% for a vector-initiated infection.

POL2FL6 isolate produced massive infections in sand flies, and both BOS1FL1 and POL2FL6 caused severe clinical manifestations in hamsters. Animals infected with the outbreak isolates exhibited high infectiousness to sand flies. Therefore, this result supports the great virulence of the two studied *Leishmania* isolates that are circulating in the focus of VL in Madrid. So far, molecular characterization of *Leishmania* isolates from the focus share the same ITS genotype as the strain MHOM/ES/87/LOMBARDI [58]. LOMBARDI strain was initially isolated in 1987 from a CL patient in Spain (region unknown). Chicharro et al. typed human *Leishmania* isolates to get an epidemiologic picture of two periods (before: 1988–2005 and during the outbreak: 2008–2012) and found out that this genotype has been circulating in Madrid since at least 1992 [58]. This is the only ITS type found in isolates from all human cases associated with this outbreak. Besides human cases, ITS-LOMBARDI is also present in field sand flies and in hares and rabbits from the focus area. This genotype has been found both in isolates from CL and VL patients [58]. Therefore, no specific tropism has been assigned to this ITS genotype. Recently, virulence of *L. infantum* isolates from *P. perniciosus* captured in the focus area was highlighted using an ex vivo model [59]. These authors showed that BOS1FL1 and POL2FL7 isolates displayed high virulence in terms of infection rates of murine macrophages and dendritic cells, cytokine production and enzymatic activities, undermining host immune defense mechanisms. The experiments included in this study with in vivo natural transmission models serve to complement the ex vivo analyses. The high virulence of these isolates is consistent with the outbreak of leishmaniasis of Madrid and could explain its magnituide, since it is considered the largest focus of VL described in Europe so far.

This natural infection model allowed us to compare *Leishmania* strain virulence and infectivity to sand flies. The virulence of isolates that are circulating in the focus of leishmaniasis in Madrid has been highlighted by the worse clinical outcome of disease, higher parasitic loads in spleen and liver and positivity of *Leishmania* serology when compared with hamsters infected with JPCM5 strain. Histopathological studies confirmed the wide spread of POL2FL6 parasites to several organs of hamsters and only animals infected with outbreak isolates were infective to sand flies in xenodiagnostic studies. These findings would contribute to a better understanding of the epidemiology of the largest focus of VL in Europe.
